# A mobile intra-abdominal low-grade fibromyxoid sarcoma arising from the jejunal mesentery in an adolescent: a case report and literature review

**DOI:** 10.3389/fped.2026.1895681

**Published:** 2026-09-30

**Authors:** Tao Lyu, Mengcheng Li, Yang Xia, Jianyin Zou, Qingchao Zhu

**Affiliations:** 1 Department of Gastrointestinal Surgery, Shanghai Sixth People’s Hospital Affiliated to Shanghai Jiao Tong University School of Medicine, Shanghai, China; 2 Department of Otorhinolaryngology Head and Neck Surgery, Shanghai Sixth People’s Hospital Affiliated to Shanghai Jiao Tong University School of Medicine, Otorhinolaryngology Institute of Shanghai JiaoTong University, Shanghai, China

**Keywords:** adolescent, case report, intra-abdominal mass, jejunal mesentery, low-grade fibromyxoid sarcoma

## Abstract

Low-grade fibromyxoid sarcoma (LGFMS) is a rare fibroblastic neoplasm that only exceptionally involves the intra-abdominal cavity. We report a 17-year-old girl with a one-year history of a progressively enlarging, painless, freely mobile abdominal mass. Computed tomography and magnetic resonance imaging showed a localized, well-circumscribed, pedunculated lesion with positional variability, initially suggesting a completely resectable gastrointestinal stromal tumor. Preoperative biopsy was not performed because systemic treatment was not anticipated and intact resection was planned while minimizing the risks of tumor rupture and intraperitoneal dissemination. A non-contrast chest computed tomography examination on admission, three days before surgery, showed no significant abnormality. Laparoscopic exploration identified an approximately 10 × 10 cm encapsulated tumor attached by a narrow peripheral stalk to the mesenteric border of the proximal jejunum, approximately 40 cm distal to the ligament of Treitz. Complete en-bloc tumor excision with segmental jejunal resection required conversion to open surgery. Histology showed bland spindle cells in alternating fibrous and myxoid stroma with low mitotic activity. The tumor was MUC4-negative, showed absent RB expression and CDK4/p16 positivity, and was essentially negative for MDM2; weak nuclear MDM2 staining was observed predominantly in vascular endothelial cells, concordant with the absence of MDM2 amplification by fluorescence *in situ* hybridization. Fusion testing was unavailable, so the diagnosis remains morphologically favored but molecularly unconfirmed. At 6 months, abdominal and chest computed tomography showed no local recurrence or metastatic disease. At 12 months, non-contrast abdominal computed tomography showed no significant abnormality or abdominal recurrence; chest imaging was not performed. The narrow peripheral mesenteric pedicle provides a directly documented anatomic explanation for the marked positional variability in this case.

## Introduction

Soft tissue sarcomas (STS) are a heterogeneous group of malignancies comprising more than 80 recognized histologic subtypes in contemporary classifications, underscoring their diagnostic complexity (1). Low-grade fibromyxoid sarcoma (LGFMS), also known as Evans tumor, was first described by Evans in 1987 (2) and remains a rare entity, accounting for approximately 0.6% of all STS, with an estimated incidence of 0.18 per million (3). LGFMS typically affects young to middle-aged adults, most commonly arising as a deep-seated mass in the extremities or trunk (4). A slight male predominance has been noted in some series (5), although others report a near-equal sex distribution (6). In the present case, the lesion had a peripheral, narrow pedicle at the mesenteric border of the proximal jejunum, approximately 40 cm distal to the ligament of Treitz.

Histologically, LGFMS is characterized by deceptively bland spindle cells arranged in a whorled or linear pattern within alternating fibrous and myxoid stroma (7). Mitotic figures and cytologic atypia are usually minimal; however, LGFMS presents a notable clinical paradox in that it may recur or metastasize many years after complete excision. Evans (5) reported local recurrences up to 15 years and metastases up to 45 years after diagnosis. Common histologic hallmarks include collagenized zones and myxoid areas, with occasional collagen rosettes, which may help distinguish LGFMS from other fibrosarcomas (8).

Accurate diagnosis of LGFMS relies on a combination of histopathology, immunohistochemistry, and molecular testing. LGFMS typically expresses vimentin and demonstrates strong MUC4 positivity (7, 9). In the cohort reported by Doyle et al. (9), all 49 FUS-rearranged LGFMSs showed MUC4 staining (49/49, 100%), which was usually diffuse and intense. At the molecular level, recurrent FUS gene rearrangements are characteristic. Most LGFMSs harbor FUS-CREB3L2, whereas less frequent alternative rearrangements include FUS-CREB3L1 and EWSR1-CREB3L1 (10, 11). Detection of these fusions provides important diagnostic support in atypical cases. Therefore, complete MUC4 negativity in an unusual anatomic site substantially lowers diagnostic certainty when confirmatory fusion testing is unavailable.

We report a freely mobile jejunal mesenteric tumor in a 17-year-old girl, with morphology favoring LGFMS but without molecular confirmation. This report documents the anatomical basis of its positional variability and reviews relevant abdominal and gastrointestinal cases.

## Literature search strategy

PubMed/MEDLINE, Crossref, and OpenAlex were searched from database inception through 19 August 2026. The search combined ‘low-grade fibromyxoid sarcoma’ or ‘Evans tumor’ with terms for abdominal and gastrointestinal sites, including abdominal/intra-abdominal, gastrointestinal, stomach/gastric, intestine/bowel, mesentery/mesenteric, mesocolon, colon, omentum, peritoneal, falciform ligament, liver, pancreas, anal, and retroperitoneal locations. The PubMed query returned 80 records before title and abstract screening. Reference lists and citing records were also screened. No language restriction was applied at the search stage; for the descriptive patient-level comparison, reports were retained when an English abstract or accessible full text permitted verification of the principal clinical variables. Primary gastrointestinal, mesenteric, peritoneal, and solid-organ abdominal tumors were considered. Abdominal-wall tumors, extra-abdominal primaries with abdominal metastasis only, duplicate reports, hybrid-only sclerosing epithelioid fibrosarcoma, and reports without a stated LGFMS or established LGFMS-spectrum diagnosis were excluded. Because heterogeneous historical reports did not permit exhaustive patient-level mapping, Table 1 presents a source-verified descriptive selection; aggregate series are summarized separately when individual variables could not be mapped.

**Table 1:** Clinical presentation, treatment, follow-up, and diagnostic evidence in selected published cases of primary intra-abdominal or gastrointestinal low-grade fibromyxoid sarcoma and the current case.

Authors (year)	Primary site	Size (cm)	Age/sex	Treatment	Follow-up	MUC4	Molecular confirmation
Kaji et al. (1999) (12)	Small-bowel mesentery	11 × 11 × 8	54/M	Mesenteric tumor + involved small-bowel resection	NED, 6 mo	NR	NR
Harish et al. (2003) (13)	Falciform ligament	21 × 16 × 10	37/M	Wide excision	NED, 12 mo	NR	NR
Koishi et al. (2003) (14)	Omentum; reported as HSCTGR (LGFMS morphologic spectrum)	NR	38/F	Excision	NR	NR	NR
Park et al. (2007) (15)	Colon, hepatic flexure; right-kidney invasion	8 (greatest dimension)	43/M	Right hemicolectomy + right nephrectomy	NED, 24 mo	NR	NR
Fujii et al. (2008) (16)	Small-bowel mesentery	17	64/M	Resection (details NR)	NR	NR	NR
Kim YJ et al. (2009) (17)	Colon, splenic flexure	10 × 6 (CT)	56/M	Colectomy + left diaphragmatic wedge resection; adjuvant radiotherapy	Regular follow-up reported; duration/status NR	NR	NR
Evans (2011) (5)	Small-bowel mesentery	15	26/M	Excision (positive margin)	Local recurrence at 3.5 and 10 y; DOD, 28 y	NR	NR
Evans (2011) (5)	Omentum/mesentery/peritoneum	9	33/M	Partial excision; repeat partial excision at 1 y; chemoradiotherapy	Progressive disease; DOD, 5 y	NR	NR
Evans (2011) (5)	Mesentery	10	39/M	Excision	Local recurrence at 2 and 18 y; alive with disease, 18.5 y	NR	NR
Laurini et al. (2011) (18)	Small intestine	14.5	53/F	Small-bowel resection	NED, 2 mo	NR	Series-level: FUS rearranged in 3/3 informative tumors; patient mapping NR
Laurini et al. (2011) (18)	Small intestine	4.5	71/M	Small-bowel resection	NED, 9 mo	NR	Series-level: FUS rearranged in 3/3 informative tumors; patient mapping NR
Laurini et al. (2011) (18)	Small intestine	NR	52/F	Small-bowel resection	DOD, 7 y	NR	Series-level: FUS rearranged in 3/3 informative tumors; patient mapping NR
Laurini et al. (2011) (18)	Small intestine	11	69/F	Small-bowel resection	Lost to follow-up	NR	Series-level: FUS rearranged in 3/3 informative tumors; patient mapping NR
Alatise et al. (2013) (19)	Proximal jejunal mesentery	30 × 26 × 14; 7.5 kg	32/M	Tumor + involved jejunoileum and transverse-colon wall resection	NED, >24 mo	NR	NR
Vasudevan and Shivamurthy (2014) (20)	Mesentery (ileocolic/ileocecal region)	11.5 × 9 × 5 (gross)	62/M	Ileocolectomy with tumor resection	Lost to follow-up after discharge	NR	NR
Kim KJ and Seo (2014); Kim M et al. (2014) (21, 22)	Transverse colon/transverse mesocolon; attached to stomach	7 × 5 × 5 (pathology; CT 6.5-7 × 4)	21/F	Transverse colectomy + partial gastrectomy	NED, 9 mo	NR	NR
Farrell et al. (2014) (23)	Transverse-colon mesentery	15 × 9 × 6 (gross; CT 15 × 10 × 7.5)	52/F	Total colectomy (abstract: subtotal colectomy)	NR	Positive, strong	Atypical FUS rearrangement by FISH
Mendoza et al. (2015) (24)	Sigmoid colon	11 × 7 × 4.5 (pathology)	48/F	Sigmoid colectomy	Postoperative bowel obstruction; oncologic follow-up NR	Positive	NR
Konecna et al. (2015) (25)	Diffuse peritoneal disease + hepatic metastasis	Diffuse nodules; liver lesion 4.9	42/F	Liver biopsy + partial omentectomy; major cytoreduction aborted/conservative management	Stable/minimal progression, 48 mo	Positive, diffuse/strong	FUS FISH noncontributory
Ban et al. (2017) (26)	External anal sphincter	6.1 × 4.6 × 4.3	27/M	Enucleation	NED, 12 mo	Positive, strong	FUS rearranged by FISH
Geramizadeh et al. (2018) (8)	Small-bowel mesentery	50 × 30	24/F	Surgical excision	NED, 6 mo	NR	Not performed (testing unavailable)
Tsukamoto et al. (2018) (27)	Stomach, lesser curvature	8.0 (greatest dimension)	54/F	Gastric wedge resection	NED, 45 mo after first visit	Positive	FUS-CREB3L1
McCarthy et al. (2019) (28)	Colon, splenic flexure	4.7	70/M	Left hemicolectomy	Pulmonary and omental metastases detected 4 y after primary resection	Positive, diffuse/strong	FUS-CREB3L1
Huang et al. (2020) (7)	Small-intestinal mesentery	10.8 × 9.0 × 8.3	43/M	Complete tumor resection	NR	Positive, diffuse/strong	FUS-CREB3L2 (FUS FISH + targeted RNA assay)
Dugalic et al. (2021) (29)	Liver	10 × 9 × 7 (imaging)	38/M	Left hepatectomy + hepatic-artery resection/reconstruction + biliary reconstruction	Spinal metastasis at 2.5 y; died at 3.5 y	Positive, diffuse/strong	NR
Beristain-Hernandez and Quintero-Sada (2021) (30)	Transverse colon; mesenteric-root infiltration	11.3 × 8.9 × 10.4 (CT); 6 × 5 × 5 (operative)	57/M	R0 extended left hemicolectomy + ascending-colon colostomy; chemotherapy (regimen NR)	Stable hepatic metastatic disease, 12 mo	NR	NR
Weiler et al. (2023) (31)	Pancreas	2.7 × 3.4 × 3.2	49/F	Open distal pancreatectomy + splenectomy	Well at 2 mo; no later follow-up arranged	Positive, diffuse/strong	NR
Barger et al. (2024) (32)	Intra-abdominal/peritoneal mass adherent to small bowel	9.0 × 6.4 × 7.7 (ultrasound); 12 (MRI)	19/F	Cesarean delivery followed by laparotomy, complete mass excision + segmental small-bowel resection	No reported recurrence under 6-monthly imaging surveillance; duration NR	NR	NR
Salinas-Castro et al. (2025) (33)	Liver	10	16/F	Partial hepatectomy	No metastasis on postoperative extension studies; duration NR	Positive, strong	NR
Al-Shurbaji et al. (2026) (34)	Stomach	NR	21/F	Incomplete laparoscopic resection; doxorubicin/ifosfamide ×6, then eribulin	Good clinical response; duration NR	NR	FUS-CREB3L1 by RT-PCR
Kirov et al. (2026) (35)	Mesentery/terminal ileum; recurrent multifocal abdomen	21 × 15 (primary)	61/F	Ileocecal resection + repeat multivisceral resections	NED, 6 mo after latest resection	Positive, strong	Not performed (unavailable)
Prochazka et al. (2026) (36)	Common bile duct	5.8 (gross)	62/F	Common-bile-duct resection + Roux-en-Y hepaticojejunostomy	NED, 7 y	Positive	NR
Lyu et al. (current case)	Peripheral proximal jejunal mesenteric stalk	10.5 × 10.2 × 5.4 (pathology)	17/F	Laparoscopic exploration converted to open laparotomy; intact en bloc tumor + segmental jejunal resection	NED, 12 mo (abdominal CT; chest CT NED at 6 mo only)	Negative	LGFMS-associated fusion testing not performed (molecularly unconfirmed); MDM2 FISH showed no amplification (differential workup only)

This descriptive selection reflects the literature search through 19 August 2026. Ages are in years and sizes in centimeters unless otherwise stated. NR indicates not reported or not verifiable from the accessible report and does not imply that a test or follow-up was absent. Kim KJ and Seo and Kim M et al. describe the same patient and were counted once. Koishi et al. reported a hyalinizing spindle cell tumor with giant rosettes, now regarded within the LGFMS morphologic spectrum. The Laurini molecular result is series-level and could not be mapped to individual patients. The current case is included as morphologically favored but molecularly unconfirmed LGFMS; MDM2 FISH nonamplification does not constitute molecular confirmation of LGFMS.

LGFMS, low-grade fibromyxoid sarcoma; CT, computed tomography; MRI, magnetic resonance imaging; RT-PCR, reverse transcription polymerase chain reaction; HSCTGR, hyalinizing spindle cell tumor with giant rosettes; DOD, died of disease; R0, resection with microscopically negative margins; mo, months; y, years; F, female; M, male; FISH, fluorescence *in situ* hybridization; NED, no evidence of disease; NR, not reported or not verifiable from the accessible source.

## Case presentation

### Clinical presentation

A 17-year-old female presented with a one-year history of a mobile abdominal mass. She first noticed a freely movable lump in her abdomen approximately one year before presentation, measuring about 3 × 3 cm. The mass was unusual in that its position appeared to “wander” within the abdominal cavity—at times it was palpable in the upper left quadrant, at other times in the upper right quadrant, and occasionally in the lower left quadrant. Over the course of the year, the mass gradually increased in size and eventually reached an estimated size of approximately 10 cm.

Despite the increasing size of the lesion, the patient reported no significant gastrointestinal symptoms. She denied abdominal pain, bloating, early satiety, nausea, vomiting, or changes in bowel habits. There was no evidence of gastrointestinal bleeding, including melena or hematochezia. Her past medical history was notable for emergency laparotomy five years earlier for traumatic liver laceration. The available outside-hospital discharge summary documented an abdominal computed tomography (CT) examination during that admission and did not report an abdominal mass. This provides an upper bound for when the current lesion became radiologically apparent but does not establish its biological onset. She had no chronic illnesses or relevant family history.

On examination, vital signs were normal and systemic examination was unremarkable. Abdominal palpation revealed a firm, well-defined, non-tender mass in the left upper abdomen measuring approximately 10 × 11 cm. The lesion was freely mobile and not adherent to the abdominal wall. The remainder of the abdominal examination was unremarkable, with no distension or additional palpable masses. Laboratory investigations, including complete blood count and metabolic panel, were within normal limits. Tumor markers for gastrointestinal and gynecologic malignancies, including carcinoembryonic antigen (CEA), CA-125, CA 15-3, CA 19-9, CA-50, and CA-242, were all within their respective reference ranges.

### Imaging findings

Outpatient contrast-enhanced magnetic resonance imaging (MRI) of the abdomen demonstrated a localized, well-circumscribed, pedunculated soft-tissue mass in the left lower abdominal cavity closely associated with the small intestine. The lesion had an apparently intact capsule, showed low signal intensity on T1-weighted sequences and heterogeneous high signal intensity on T2-weighted sequences (Figures 1A–C), and exhibited heterogeneous enhancement after intravenous gadolinium administration (Figure 1D; Figures 2A, B). No definite invasion of adjacent organs or peritoneal dissemination was identified.

**Figure 1:**
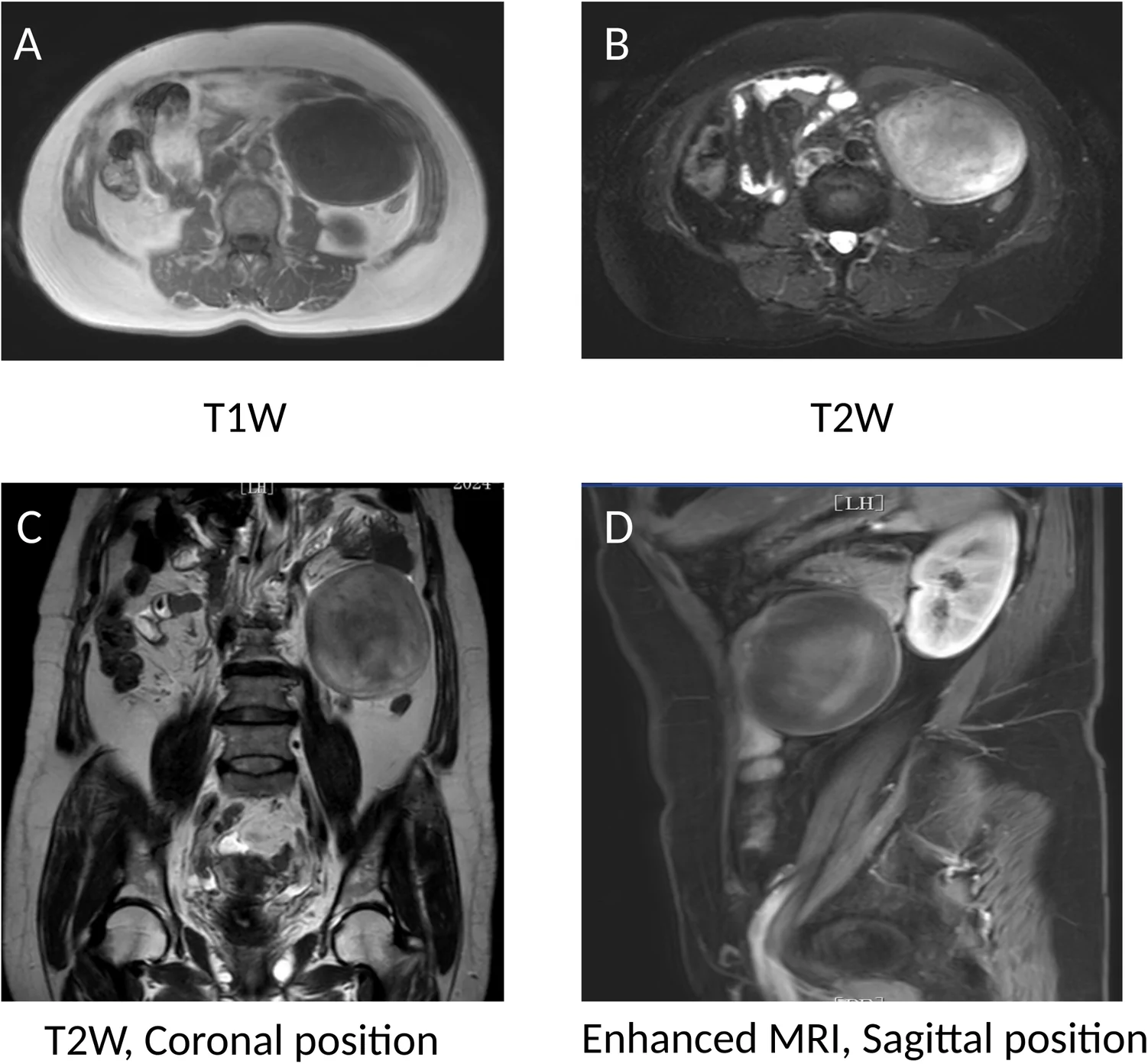
Preoperative magnetic resonance imaging of a mobile jejunal mesenteric tumor in a 17-year-old girl. Preoperative contrast-enhanced magnetic resonance imaging (MRI) of the abdomen demonstrating a large soft-tissue mass closely associated with the small intestine in the left lower abdominal cavity. **(A)** Axial T1-weighted (T1W) image showing predominantly low signal intensity of the lesion. **(B,C)** Axial **(B)** and coronal **(C)** T2-weighted (T2W) images demonstrating heterogeneous high signal intensity. **(D)** Contrast-enhanced image demonstrating heterogeneous enhancement within the mass.

**Figure 2:**
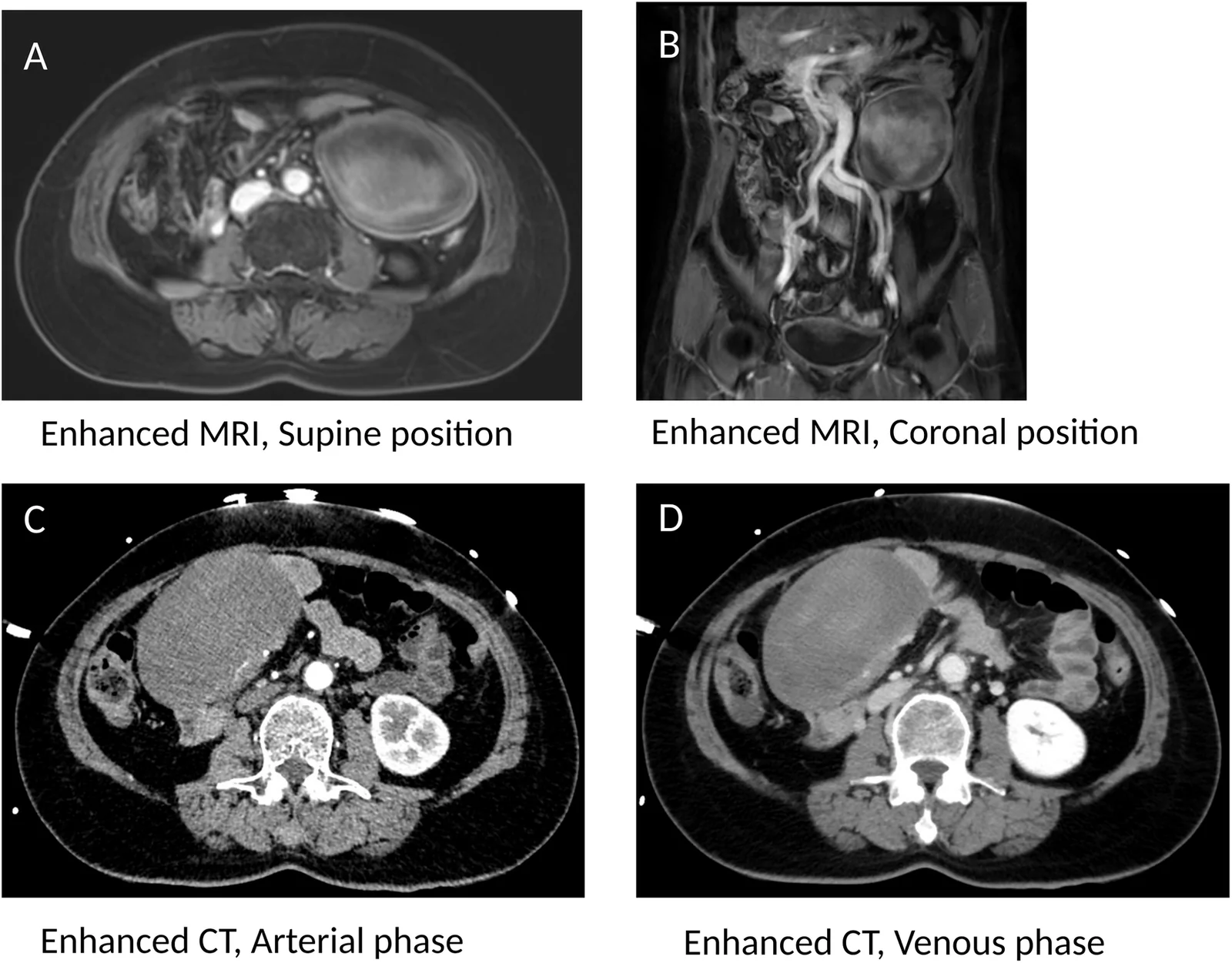
Positional change of a jejunal mesenteric tumor between preoperative magnetic resonance imaging and computed tomography in a 17-year-old girl. Serial imaging demonstrating positional variability of the intra-abdominal mass. **(A,B)** Outpatient contrast-enhanced magnetic resonance imaging (MRI), in axial **(A)** and coronal **(B)** planes, showing the lesion located in the left lower abdominal cavity adjacent to the small intestine. **(C,D)** Contrast-enhanced abdominal computed tomography (CT) in arterial **(C)** and venous **(D)** phases, performed on the second hospital day following admission one week after outpatient MRI, demonstrating the same mass in the right upper abdomen.

The radiologic differential diagnosis included a pedunculated small-intestinal gastrointestinal stromal tumor (GIST) and a mesenteric fibrous tumor. Because the lesion appeared localized and completely resectable, preoperative systemic treatment was not anticipated. A preoperative biopsy was therefore not obtained, and the team proceeded directly to surgery to permit intact resection while minimizing the potential risks of tumor rupture and intraperitoneal dissemination associated with tissue sampling (37). If imaging had suggested unresectable, marginally resectable, or multifocal disease, image-guided core-needle biopsy with histologic and molecular characterization would have been considered before systemic or neoadjuvant treatment planning.

The patient was admitted one week after the outpatient MRI for laparoscopic exploration and tumor resection. A non-contrast chest CT examination performed on admission, three days before surgery, showed no significant abnormality. On repeat examination, the palpable mass had migrated to the upper right abdomen, consistent with its previously observed mobility. To reassess tumor location and characteristics, a follow-up contrast-enhanced abdominal CT scan was performed on the second hospital day. CT confirmed a large, well-circumscribed mass with heterogeneous enhancement, now located in the right upper abdomen. No adjacent-organ invasion, peritoneal dissemination, or new lesion was identified (Figures 2C, D).

### Surgical findings and procedure

The patient underwent surgical exploration under general anesthesia on the fourth hospital day. Initial diagnostic laparoscopy revealed an encapsulated, smooth-surfaced mass measuring approximately 10 × 10 cm arising from the mesenteric border of the proximal jejunum, approximately 40 cm distal to the ligament of Treitz (Figures 3A–C). The tumor was attached to the small bowel by a peripheral, narrow mesenteric stalk and was free of adhesions to surrounding organs. The peripheral pedicle and this local absence of adhesions explained the remarkable mobility of the mass. The adjacent bowel serosa appeared intact, and there was no evidence of intestinal obstruction or significant proximal bowel dilatation.

**Figure 3:**
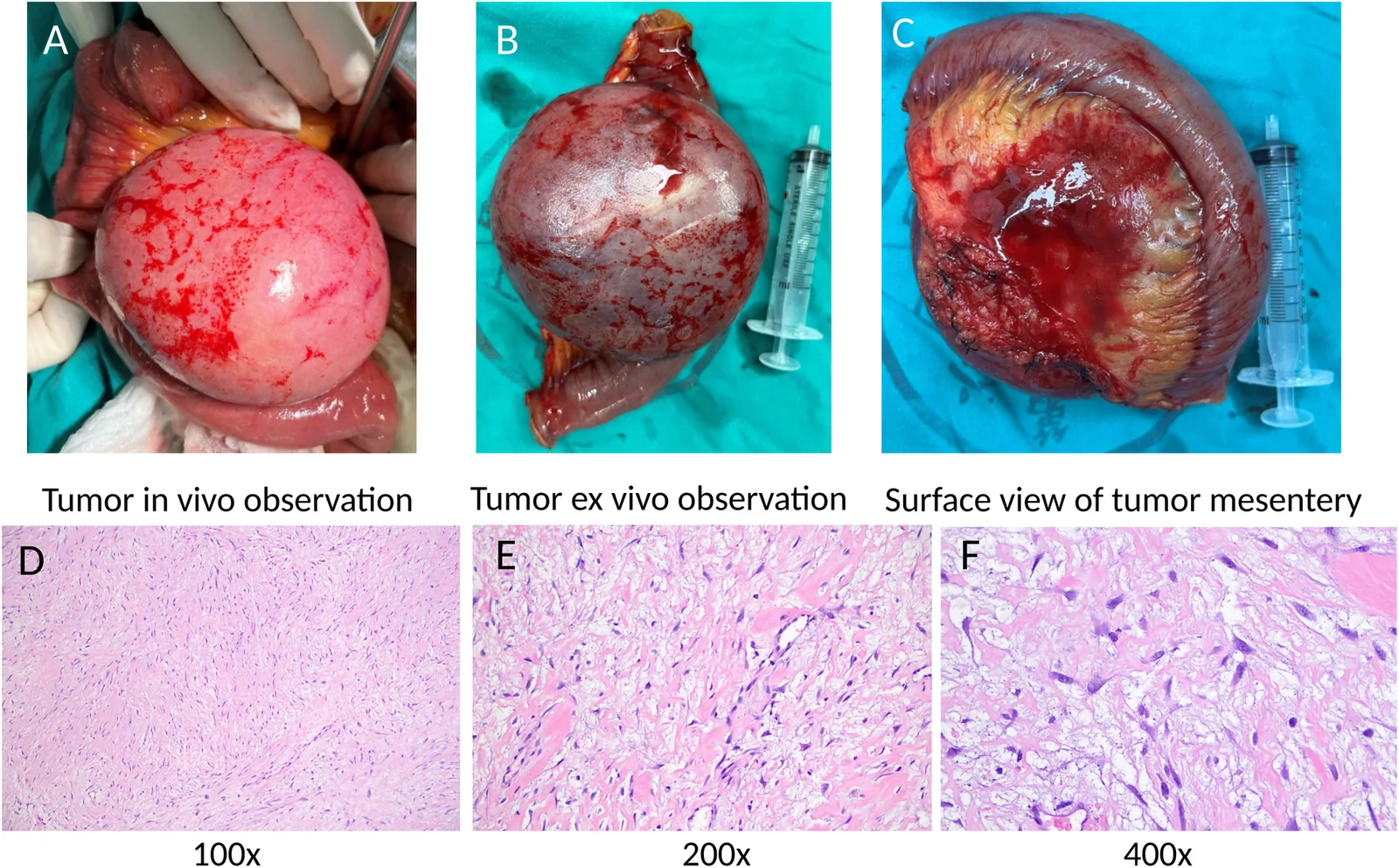
Operative, gross, and histological features of a jejunal mesenteric tumor morphologically favoring low-grade fibromyxoid sarcoma in a 17-year-old girl. **(A)** Intraoperative *in vivo* view demonstrating a large, smooth, well-circumscribed, encapsulated mass arising from the jejunal mesentery. **(B)** Gross *ex vivo* appearance of the resected specimen following complete surgical excision. **(C)** Surface view of the mesenteric attachment of the tumor after resection, demonstrating origin from the mesenteric border of the jejunum. **(D)** Hematoxylin and eosin staining at low magnification (100×) showing hypocellular spindle-cell proliferation within collagenous stroma with focal myxoid change. **(E)** Intermediate magnification (200×) demonstrating bland spindle cells arranged in fascicular and whorled patterns. **(F)** High magnification (400×) showing uniform spindle cells with minimal cytologic atypia and low mitotic activity, morphologically favoring low-grade fibromyxoid sarcoma; fusion testing was unavailable.

Because the approximately 10 cm mass could not be retrieved safely through the laparoscopic access without compression, capsular breach, or spillage, the procedure was converted to a midline laparotomy. The mass was delivered intact and resected en bloc with the involved jejunal segment and corresponding mesentery, including approximately 5 cm of grossly normal bowel proximally and distally. A primary side-to-side jejunal anastomosis was performed. The procedure was completed without tumor rupture or spillage, and gross margins were negative.

### Histopathology and immunohistochemistry

Gross examination of the resected specimen revealed a well-circumscribed mass measuring 10.5 × 10.2 × 5.4 cm attached to a segment of small intestine. The tumor was located on the serosal surface of the jejunum and was enclosed by an intact smooth capsule. The cut surface was gray-white, solid, and firm. Importantly, the tumor did not invade the muscularis propria or mucosal layers of the small intestine. All surgical margins, including the proximal and distal intestinal margins and mesenteric margin, were free of tumor.

Histologically, the lesion was composed of bland spindle-shaped cells arranged in whorled and long fascicular patterns within collagenous stroma with extensive myxoid change (Figures 3D–F). Occasional thick collagen bundles were present. Cytologic atypia was minimal and mitotic activity was very low. These findings morphologically favored LGFMS.

Immunohistochemical studies demonstrated a low proliferative index, with Ki-67 approximately 3%. Tumor cells were negative for CD117, DOG1, and CD34, arguing strongly against GIST (Figure 4). Markers of smooth muscle and neural differentiation, including desmin, smooth muscle actin (SMA), and S-100, were also negative. Epithelial markers (pan-cytokeratin and AE1/AE3) and progesterone receptor were negative (Supplementary Figure S1). RB protein expression was absent.

**Figure 4:**
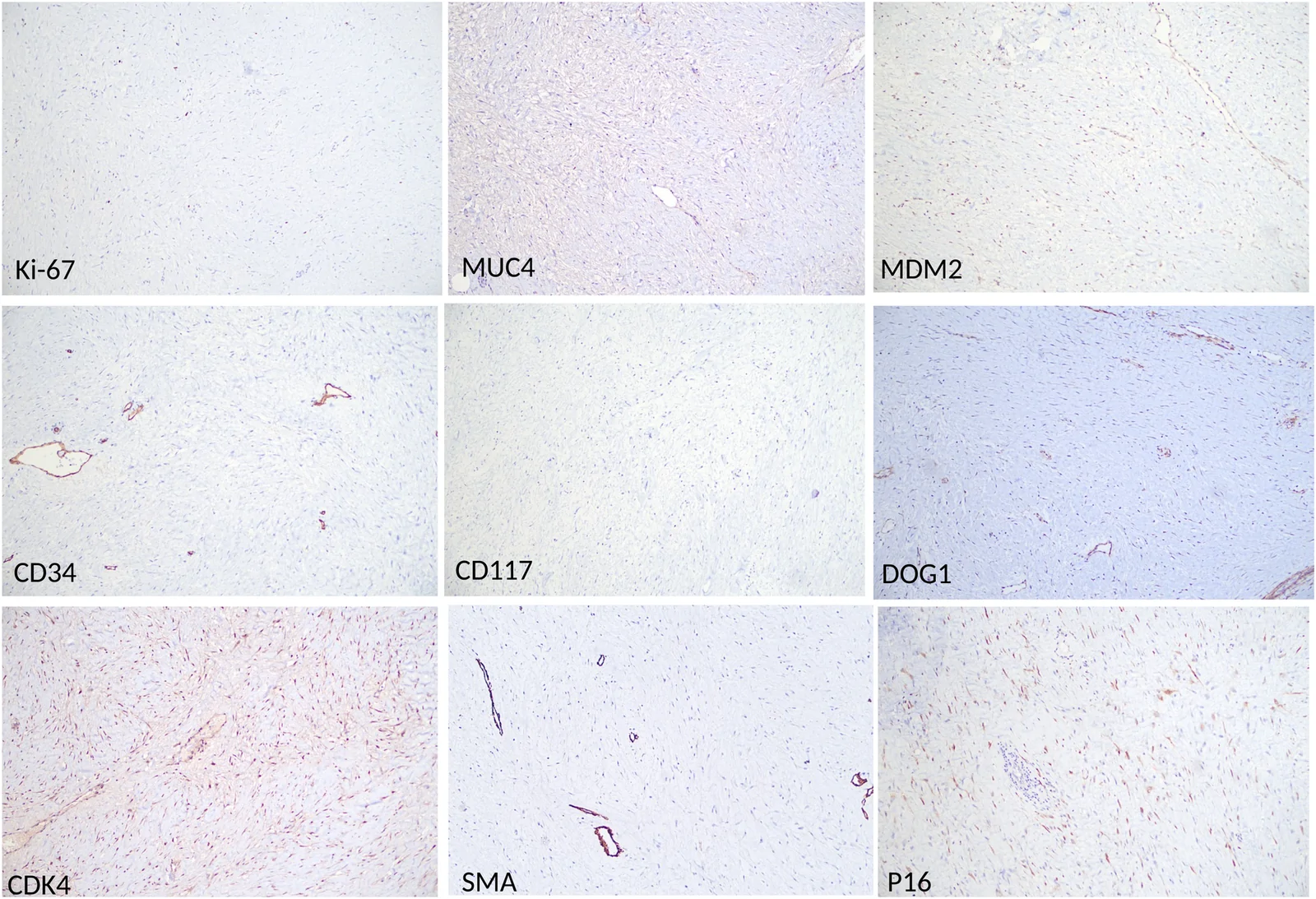
Immunohistochemical findings in a MUC4-negative jejunal mesenteric tumor morphologically favoring low-grade fibromyxoid sarcoma in a 17-year-old girl. Representative immunohistochemical findings, arranged from left to right. Top row: Ki-67 proliferative index approximately 3%; absent MUC4 expression; weak nuclear MDM2 staining predominantly in vascular endothelial cells, with tumor cells essentially negative. Middle row: tumor cells were negative for CD34, CD117 (c-KIT), and DOG1. Bottom row: CDK4 and p16 were positive; smooth muscle actin (SMA) was negative. RB protein expression was absent and MDM2 fluorescence *in situ* hybridization (FISH) showed no gene amplification (both not shown).

MUC4 was negative, while tumor cells showed CDK4 and p16 positivity and absent RB expression. Weak nuclear MDM2 staining was observed predominantly in vascular endothelial cells, whereas the tumor cells were essentially negative for MDM2. This staining pattern was concordant with the absence of MDM2 amplification by fluorescence *in situ* hybridization (FISH). Archived tissue remains available; however, LGFMS-associated fusion testing was not available at our institution and was therefore not performed during this revision. Two pathologists re-reviewed the histomorphology and immunohistochemical findings and considered the morphology most consistent with LGFMS. Given the atypical immunophenotype and absence of fusion confirmation, the diagnosis remains morphologically favored but molecularly unconfirmed.

### Postoperative course

The patient's postoperative recovery was uneventful. Bowel function returned on postoperative day 2, with passage of flatus and independent ambulation. Oral intake was resumed with a liquid diet on postoperative day 3 and advanced to a soft diet by postoperative day 4. By postoperative day 4, inflammatory markers and complete blood count remained within normal limits. The abdominal drainage tube was removed on postoperative day 7 after normalization of bowel function.

The patient was discharged in stable condition on postoperative day 7 with plans for regular outpatient follow-up and imaging surveillance.

### Follow-up

At 6 months after surgery, abdominal and chest CT showed no evidence of local recurrence or metastatic disease. At the latest documented follow-up, 12 months after surgery, non-contrast abdominal CT showed no significant abnormality or abdominal recurrence. Chest imaging was not performed at that visit. The next follow-up is scheduled for 24 months after surgery and will include non-contrast abdominal CT; no chest imaging is currently scheduled. The treating team will re-evaluate whether to include chest imaging before that visit in light of the recognized risk of late pulmonary metastasis. Later surveillance timing and modalities will be individualized after the 24-month assessment.

### Timeline

The clinical course and planned follow-up are summarized in Table 2.

**Table 2:** Clinical course, diagnostic assessment, treatment, and planned follow-up of a 17-year-old girl with a mobile jejunal mesenteric tumor morphologically favoring low-grade fibromyxoid sarcoma.

Time	Clinical event
Five years before the current presentation	Emergency laparotomy for traumatic liver laceration at an outside hospital. The available discharge summary documented abdominal CT and did not report an abdominal mass.
Approximately one year before presentation	The patient first noticed a freely mobile abdominal lump measuring approximately 3 × 3 cm.
At presentation/outpatient assessment	A painless, mobile mass measuring approximately 10 × 11 cm was found on abdominal palpation. Outpatient contrast-enhanced MRI localized the lesion to the left lower abdomen.
Admission, one week after outpatient MRI (hospital day 1)	The mass was palpable in the right upper abdomen. Non-contrast chest CT on admission showed no significant abnormality.
Hospital day 2	Contrast-enhanced abdominal CT confirmed the mass in the right upper abdomen, demonstrating positional migration.
Hospital day 4 (surgery)	Laparoscopic exploration identified an approximately 10 cm tumor attached by a narrow stalk to the proximal jejunal mesenteric border. Conversion to midline laparotomy permitted intact en bloc tumor and segmental jejunal resection without rupture or spillage.
Postoperative pathologic assessment (date not specified)	Morphology favored LGFMS. The atypical immunophenotype and unavailable LGFMS-associated fusion testing left the diagnosis molecularly unconfirmed; MDM2 FISH showed no amplification.
Postoperative days 2-7	Bowel function returned, oral intake was advanced, and the patient was discharged in stable condition on postoperative day 7.
6 months after surgery	Abdominal and chest CT showed no local recurrence or metastatic disease.
12 months after surgery	Non-contrast abdominal CT showed no significant abnormality or abdominal recurrence; chest imaging was not performed.
24 months after surgery (planned)	Non-contrast abdominal CT is scheduled. Chest imaging is not currently scheduled and will be re-evaluated before the visit.

Admission is hospital day 1. Time points are relative to presentation, admission, or surgery as stated and do not establish biological tumor onset. The diagnosis remains molecularly unconfirmed. Two pathologists re-reviewed the tumor during the preceding manuscript revision. The 24-month visit is planned.

CT, computed tomography; MRI, magnetic resonance imaging; LGFMS, low-grade fibromyxoid sarcoma; FISH, fluorescence *in situ* hybridization.

### Patient perspective

A patient-authored perspective was not available.

## Discussion and literature review

### Epidemiology and anatomical distribution

LGFMS is an uncommon soft-tissue sarcoma that typically arises in deep soft tissues of the extremities and trunk of young adults (4). Abdominal and gastrointestinal occurrences are rare. A 2018 clinicopathologic study described 13 abdominopelvic and retroperitoneal cases; all were MUC4-positive, and all five tumors tested for FUS rearrangement were positive (38). A 2026 series of 26 abdominopelvic and retroperitoneal tumors reported an age range of 5–75 years, diffuse MUC4 positivity in all cases, and FUS or EWSR1 rearrangements in all nine tested tumors (39).

Selected source-verified patient-level reports of primary intra-abdominal or gastrointestinal LGFMS are summarized in Table 1. The literature includes a MUC4-positive primary hepatic LGFMS in a 16-year-old girl (33), and the aggregate 2026 series included patients as young as 5 years (39), although the individual site of the youngest patient was not available from the aggregate report. The present 17-year-old patient therefore should not be described as the youngest reported abdominal case. The distinctive contribution of the present report is the direct operative demonstration that a narrow jejunal mesenteric pedicle accounted for the position changes documented on serial examination and imaging.

Within the gastrointestinal tract, LGFMS has been reported in the small intestine, colon, stomach, external anal sphincter, and common bile duct, while other intra-abdominal tumors have arisen in the mesentery, mesocolon, omentum, falciform ligament, liver, and pancreas (Table 1). Laurini et al. (18) reported four small-intestinal cases with molecular cytogenetic confirmation. Aggregate abdominopelvic and retroperitoneal series are discussed separately because patient-level variables could not be mapped reliably (38, 39).

Tumor size in this region is often substantial at presentation. Several reports have described large abdominal lesions, including a 50 × 30 cm mesenteric tumor (8) and a 21 cm falciform-ligament tumor (13). These observations indicate that LGFMS can rarely arise in gastrointestinal or intra-abdominal sites and should be considered in the differential diagnosis of a deep-seated abdominal spindle-cell neoplasm.

### Clinical presentation and imaging

Patients with gastrointestinal or intra-abdominal LGFMS often present with nonspecific symptoms or are diagnosed incidentally. Many tumors are discovered as a palpable abdominal mass or during imaging performed for unrelated reasons. Common presentations include abdominal discomfort, abdominal distension, or a slowly enlarging abdominal mass. When the tumor arises from the bowel wall, gastrointestinal bleeding, obstruction, or intussusception may occur, although these presentations are uncommon.

Marked position changes have also been documented in a pregnancy-associated abdominal LGFMS, in which the mass moved from the right to the left abdomen in the setting of an enlarging uterus and suspected torsion (32). The present case is distinguished by paired imaging and operative demonstration that a narrow peripheral pedicle at the proximal jejunal mesenteric border provided the mechanical basis for marked mobility. Despite the prior laparotomy, the tumor itself was free of adhesions to surrounding organs at the current operation, which further permitted its movement.

On CT, LGFMS usually appears as a well-defined soft-tissue mass with heterogeneous internal density. Larger lesions may contain fibrous and myxoid components and can appear lobulated or partially low attenuation. These tumors often displace adjacent bowel or abdominal organs without obvious invasion, consistent with their indolent growth pattern (25).

The radiologic differential diagnosis includes GIST, desmoid-type fibromatosis, liposarcoma, solitary fibrous tumor, and other spindle-cell neoplasms. In a tumor with absent RB expression, the pathologic differential also includes RB1-deficient mesenchymal neoplasms, particularly atypical spindle cell/pleomorphic lipomatous tumor (40). Imaging findings are nonspecific, and definitive classification requires integration of histomorphology, immunohistochemistry, and, when available, molecular testing.

Accordingly, the present tumor is reported as morphologically favored LGFMS that remains molecularly unconfirmed, rather than as a fusion-confirmed example.

### Histopathology and immunophenotype

LGFMS is characterized by deceptively bland morphology. Tumors are composed of uniform spindle to ovoid cells with minimal atypia and low mitotic activity, arranged in fascicular, whorled, or storiform patterns (7, 41). A characteristic feature is alternating fibrous and myxoid stroma, with collagen-rich hypocellular areas merging with more cellular myxoid regions. Giant collagen rosettes may also be present in a subset of cases.

Intra-abdominal LGFMS can occasionally demonstrate unusual morphologic patterns. Huang et al. (7) described a mesenteric LGFMS with epithelioid whorls and diffuse DOG1 expression, illustrating a potential diagnostic pitfall. Nevertheless, the defining histologic features remain bland spindle-cell proliferation, alternating fibrous–myxoid architecture, and very low mitotic activity.

Immunohistochemically, MUC4 is a highly sensitive and specific marker for LGFMS and is usually diffuse and strong (9). The two abdominopelvic and retroperitoneal series also reported diffuse MUC4 positivity in all tumors (38, 39). Complete MUC4 negativity in the present case therefore represents a substantial departure from the expected immunophenotype.

Weak or equivocal MDM2 immunoreactivity can be misleading because MDM2 immunohistochemistry is less specific than amplification testing (42). In the present tumor, weak nuclear staining was predominantly endothelial and the tumor cells were essentially negative; this pattern was concordant with the absence of MDM2 amplification by FISH and did not support an MDM2-amplified liposarcoma.

Two pathologists re-reviewed the case and considered the bland spindle-cell morphology with alternating fibrous and myxoid stroma most consistent with LGFMS. Nevertheless, the MUC4-negative, RB-loss, and CDK4/p16-positive phenotype is atypical, and LGFMS-associated fusion testing was not performed. These limitations necessitate the qualified diagnosis used throughout this report.

### Molecular pathology

LGFMS is characterized by recurrent chromosomal rearrangements involving the FUS gene. The most common fusion is FUS–CREB3L2, while a smaller subset demonstrates FUS–CREB3L1 or EWSR1–CREB3L1 fusion (10, 11).

Detection of these fusions is particularly useful in diagnostically challenging cases, including unusual anatomic sites or atypical immunophenotypes. FISH, reverse transcription polymerase chain reaction (RT-PCR), and targeted RNA sequencing have all been used to confirm the diagnosis (7, 10, 18).

In the present case, archived tissue remains available, but LGFMS-associated fusion testing was not available at our institution and therefore was not performed during this revision. MDM2 FISH showed no amplification, but this finding addresses an MDM2-amplified differential diagnosis and does not confirm LGFMS. The absence of fusion confirmation is therefore a central diagnostic limitation.

### Treatment and prognosis

Complete surgical excision remains the mainstay of treatment for LGFMS (1, 6). This is also true for gastrointestinal and intra-abdominal lesions. When feasible, en bloc resection with clear margins offers the best chance for durable disease control. There is currently no established role for chemotherapy or radiotherapy in completely resected localized LGFMS. Available evidence suggests limited sensitivity to systemic chemotherapy, even in advanced disease (6).

LGFMS has a paradoxical clinical course: short-term outcomes are often favorable, but local recurrence and distant metastasis may occur many years or even decades after resection (5, 6). The lungs are the dominant metastatic site, although liver, pleura, bone, and peritoneal metastases have also been reported.

There is no unified evidence-based surveillance schedule specific to LGFMS. Follow-up was therefore individualized using general soft-tissue sarcoma guidance as a framework together with tumor location, recurrence risk, and local practice (1). The next assessment is scheduled at 24 months with non-contrast abdominal CT. Chest imaging is not currently included, but the treating team will re-evaluate its inclusion before that visit given the risk of late pulmonary metastasis. Subsequent timing and modalities will be individualized, with long-term surveillance emphasized.

Strengths of this report include serial imaging and operative documentation that directly link the tumor's mobility to a narrow peripheral mesenteric pedicle. Limitations include the atypical immunophenotype, absence of fusion confirmation and 12-month documented follow-up. A mobile, well-circumscribed mesenteric spindle-cell tumor may mimic GIST; intact resection and cautious, explicitly qualified pathologic interpretation are essential when classic morphology is not supported by the expected immunophenotype or molecular confirmation.

## Data Availability

The original contributions presented in the study are included in the article/Supplementary Material, further inquiries can be directed to the corresponding authors.

## Supplementary material

SUPPLEMENTARY FIGURE S1 Additional immunohistochemical findings in a jejunal mesenteric tumor morphologically favoring low-grade fibromyxoid sarcoma in a 17-year-old girl. Representative immunohistochemical staining demonstrating negative desmin, S100, cytokeratin (CK), and progesterone receptor (PR). These findings help exclude selected morphologic mimics but do not independently establish low-grade fibromyxoid sarcoma.
